# The Impact of Elastic Deformations of the Extracellular Matrix on Cell Migration

**DOI:** 10.1007/s11538-020-00721-2

**Published:** 2020-04-04

**Authors:** A. A. Malik, B. Wennberg, P. Gerlee

**Affiliations:** Department of Mathematical Sciences, Chalmers University of Technology and University of Gothenburg, 412 96 Gothenburg, Sweden

**Keywords:** Durotaxis, Mathematical modeling, Stochastic simulation, Cell migration

## Abstract

The mechanical properties of the extracellular matrix, in particular its stiffness, are known to impact cell migration. In this paper, we develop a mathematical model of a single cell migrating on an elastic matrix, which accounts for the deformation of the matrix induced by forces exerted by the cell, and investigate how the stiffness impacts the direction and speed of migration. We model a cell in 1D as a nucleus connected to a number of adhesion sites through elastic springs. The cell migrates by randomly updating the position of its adhesion sites. We start by investigating the case where the cell springs are constant, and then go on to assuming that they depend on the matrix stiffness, on matrices of both uniform stiffness as well as those with a stiffness gradient. We find that the assumption that cell springs depend on the substrate stiffness is necessary and sufficient for an efficient durotactic response. We compare simulations to recent experimental observations of human cancer cells exhibiting durotaxis, which show good qualitative agreement.

## Introduction

Cell migration is essential to many processes such as embryogenesis (Kurosaka and Kashina 2008) and wound healing (Parkin and Cohen 2001), but is also important in many diseases, such as cancer (Wang et al. 2005; Yamaguchi et al. 2005). Two common mechanisms for cell locomotion are “swimming” and “crawling.” The typical example of swimming motion is that of *E*. *Coli*. It alternates between moving in a more or less straight path for a random duration of time and tumbling to reorient with a negligible change in spatial position. The second type of locomotion is crawling, where the cell extends protrusions, formed by the cytoskeleton, a fiber network within the cell consisting of protein filaments. These protrusions adhere to the extracellular matrix, and the cell pulls itself forward. This is the type of motion we consider in this study. It is a cyclic process that can be conceptually described as consisting of four phases (Kurosaka and Kashina 2008). The first is the polarization phase, followed by the protrusion phase, in which the cytoskeleton changes shape by extending a protrusion at the leading edge, which is driven by actin polymerization. The third phase is the attachment phase during which it adheres to the substrate on which it is crawling. The last phase is the retraction phase, where the cell pulls itself forward (Alberts 2017; Kurosaka and Kashina 2008), and adhesion sites at the back end detach.

It is known that both the chemical and mechanical properties of the microenvironment influence cells in many different ways, e.g., proliferation, differentiation, migration and the survival of cells (Keogh et al. 2010; Murphy et al. 2012). The non-cellular component of tissues and organs in the human body is called the extracellular matrix (ECM). It consists mostly of proteoglycans and fibrous proteins such as collagen and elastin (Frantz et al. 2010). The ECM serves as a scaffolding for cells, and since cells can adhere to the ECM, it facilitates migration. Mechanical properties of the ECM are known to influence cell migration, e.g., the fiber density (Kaufman et al. 2005; Sander 2014), as well as fiber orientation (Schwarz and Bischofs 2005), and cell–substrate adhesiveness (Carter 1967). Another mechanical property relevant to cell migration is the stiffness of the ECM. It has been shown that a spatially varying stiffness can result in directed cell migration up stiffness gradients, a phenomenon referred to as durotaxis (Lo et al. 2000).

### Durotaxis

The term durotaxis was first used in the study by Lo et al. (2000), but has since been observed in many different cell types (Kuboki et al. 2014; Isenberg et al. 2009; Joaquin et al. 2016; DuChez et al. 2019). In the original experiment (Lo et al. 2000), fibroblasts were cultured on a flexible polyacrylamide sheet coated with type I collagen. The sheet had a soft side and a rigid side, and cells were migrating across the boundary. The experiment showed that when cells approached the boundary from the soft side, they moved across it into the region of higher stiffness. When the cells moved from the region of high stiffness, the protrusion crossing into the softer side stopped and cells did not move in to the softer side. The authors proposed that the phenomenon was due to a mechanism where cells sense small changes in stress and strain in the substrate, which is translated into increased traction forces causing a bias in movement direction. An alternative explanation that the authors do not support is that durotaxis is the result of substrate deformations (Lo et al. 2000). If there is a gradient in substrate stiffness, the forces exerted by the cell give rise to a skewed mass distribution of the substrate, which might lead to durotaxis, as has been investigated in a computational study (Escribano  et al. 2018).

An insightful study was recently conducted (DuChez et al. 2019) which showed that multiple types of human cancer cells (two glioblastomas, metastatic breast cancer, and fibrosarcoma) exhibit durotaxis when they migrate on a substrate with a stiffness gradient. The migratory behavior of the cells on the substrate with a stiffness gradient was compared to that of cells placed on substrates with uniform stiffness. Measurements included a durotactic index called forward migration index (FMI), persistence and migration speed. Among their findings was that durotaxis occurred most efficiently on regions where the local stiffness was low, compared to regions with higher stiffness when the gradient was the same. A correlation analysis was also performed which showed that persistence (straightness) contributed little to the migration up the stiffness gradient, and that cell speed was not at all correlated with migration up the gradient.

The aim of this study is to analyze how deformations of the substrate influence cell migration with and without a stiffness gradient. We do that by formulating a model of single cell migration, which is coupled to an elastic extracellular matrix that deforms under the forces exerted by cells. To validate our model, we choose parameters that match those of the cells investigated in DuChez et al. (2019) and compare a number of measures, including forward migration index (FMI) and cell migration speed.

### Existing Mathematical Models of Cell Migration

Mathematical models of cell migration exist in many forms. Some focus on the subcellular processes such as dynamics of protrusions and stress fibers, or formation of adhesion sites (Kim et al. 2015, 2012; Harland et al. 2011). At a larger spatial scale, the entity of interest is often individual cells, such as in the model of Chaplain and coworkers (Schlüter et al. 2012). In their individual-based model, the cells were able to exert forces on the ECM resulting in realigned matrix fibers, which were modeled as thin cylinders. Their model showed two interesting properties: the first being that cells had a slight preference for stiffer regions, and the second being that the migration speed was lower on very stiff matrices. One can also describe cells at a population level. This is often done by means of partial differential equations (PDEs) describing the temporal change in the density of cells. An example of such a model is the fluid-type model developed by Dyson et al. (2016). They model collagen, culture medium and cells as a three-phase mixture, resulting in a system of partial differential equations for the respective densities. The medium and cells were assumed to be isotropic viscous fluids, and the matrix was assumed to be a transversely isotropic incompressible viscous fluid. Other examples of PDE-type models of migrating cells include Hillen’s model (Hillen 2006). This model was used to investigate migration in directed and undirected tissue networks, and it was assumed that the network fibers could be degraded by cells. Fibers orthogonal to the cell direction were more likely to be degraded than fibers parallel to the direction of movement. A similar model was later developed that investigated different migration strategies of amoeboid cells and for mesenchymal cells (Painter 2009). Amoeboid migration was described as having high velocity with a frequent change in direction and with little matrix remodeling. Mesenchymal migration was assumed to have lower velocity, less frequent direction changes, with major matrix remodeling.

A number of models have been developed with the aim of deepening the understanding of durotaxis. The Cellular Potts model is a versatile type of model that has been used (van Oers et al. 2014; Allena et al. 2016; Rens and Merks 2019). The model in Allena et al. (2016) is a Cellular Potts model used for single cell migration, where the Hamiltonian is constructed to account for cell adhesion and cell shape. A wide range of different matrix designs were used and compared to experimental observations, which were shown to be in good qualitative agreement. The Cellular Potts model can also be combined with finite element models of the ECM (van Oers et al. 2014; Rens and Merks 2019). In van Oers et al. (2014) the ECM was described as a linearly elastic and isotropic medium, and cells were able to deform the matrix by exerting forces. In their model, they assumed strain stiffening of the ECM, i.e., that it is stiffer along the orientation of strain than perpendicular to it. Durotaxis was explicitly included in their cellular Potts description through an additional term in the Hamiltonian. The extra term caused cells to have a higher probability to extend protrusions in the direction of higher stiffness and reduced probability of retraction in that direction. The model was recently extended (Rens and Merks 2019) to include an explicit description of the adhesion dynamics. It is shown that the model exhibits durotaxis as a result of two assumptions: firstly that cells can build up forces faster on stiff matrices and secondly that matrix stresses reinforces cell–matrix adhesions.

Another group of models of durotaxis are those based on random walks (Novikova et al. 2017; Doering et al. 2018; Stefanoni et al. 2011; Malik and Gerlee 2019). It is typically the position of a cell that undergoes random motion, either through random changes in position or through random changes in velocity. In these types of models, there are multiple ways to model the cells’ ability to sense its environment, which have slightly different outcomes on model predictions. This was investigated for a persistent random walk in Doering et al. (2018) where different types of stiffness sensing were investigated, with the purpose of distinguishing which mechanism is most efficient for transporting a population of cells up a gradient.  Stefanoni et al. (2011) proposed a 2D numerical model consisting of a modified version of the Langevin equation, where the stochastic force depends on the local stiffness. They assume that cells are able to sense the local stiffness through the magnitude of deformations, which are smallest in the direction of highest stiffness. The angular distribution is modified so that cells are more likely to pick a direction where the stiffness is high, but the stiffness does not impact the radial distribution. They compare their model to the experiments’ results of Lo et al. (2000) and found good agreement. Although their model exhibits durotaxis, it offers no insight into its underlying mechanism. An alternative to stochastic models of random motion is to use the corresponding transport equation, as was done in Loy and Preziosi (2019). The authors allowed for a so-called double bias where the external environment may influence both the direction of motion and the speed of the cells, and durotaxis was studied as a special case. Another possible explanation for durotaxis was investigated in Malik and Gerlee (2019), where we investigated how the longer lifetime of adhesion sites on stiff matrices can induce durotaxis, and showed that our model was able to reproduce cell clustering on a matrix with a periodically varying stiffness profile, similar to what has been observed in experiments.

The matrix stiffness is believed to impact cells in multiple ways, including the ability to generate traction forces, the dynamics of focal adhesions as well as cell shape and size (Dembo and Wang 1999; Reinhart-King et al. 2005, 2008; Califano and Reinhart-King 2010). Because of the ability of the ECM to deform under the forces generated by cells, the issue becomes even more complicated as feedback may take place, which can influence the cell itself or its neighbors. Because of that, it may be useful to isolate a single mechanism at a time for study using mathematical models. One such example is the impact of elastic deformations of the ECM on cell migration. This was part of a recent computational model (Escribano  et al. 2018), which studied single and collective cell migration in a linearly elastic substrate with different stiffness gradients. The cell was made up of three parts: the first part being a central contractile rod; the second part made up of adhesive zones on either side of the central part, which adhere to the ECM; and the third being the protrusive part, located at the outer edges of the cell. The authors observed that durotaxis resulted from the substrate being deformed more at the soft end of the cell, as well as cell growing faster on the stiffer side of the cell. They showed that larger cell monolayers, representing collective cell migration, more efficiently migrate up the stiffness gradient. The model developed is complex and contains many different interacting parts, including both deformation of the ECM as well as the dynamic binding/unbinding of adhesion complexes and cell growth.

In this work, we have focused on the impact of substrate deformation alone, and develop a simpler model. We assume that cells move randomly by extending protrusions which adhere to the substrate and exert forces so that it deforms elastically in response to the forces. The model we consider is simplified compared to the actual dynamics of cell migration, but provides a step toward understanding how substrate deformations influence cell migration. We first investigate substrates of varying stiffness, where the stiffness in each case is spatially homogeneous. We then introduce a gradient in the stiffness and find from the stochastic simulations that this results in a bias in the direction of increasing stiffness. As it is known that cells tend to generate larger traction forces when the substrate is stiff, we also distinguish between two different cases for how cells generate forces, the first being independent of substrate stiffness and the second where traction forces are proportional to substrate stiffness.

## Model Formulation

In this section, we describe the mathematical model of a migrating cell and the model for the extracellular matrix. In what follows, we assume that the cell migration takes place in 1D.

### Model of a Cell

Our mathematical model of a cell is a simplified version of the cell model introduced by Dallon et al. (2013b, 2013a), in which a cell is assumed to consist of a nucleus with position $\mu$, attached to *n* adhesion sites at positions $X_i$, $i = 1,2,\ldots , n$, connected to the nucleus with elastic springs of rest length 0, with spring coefficients $\alpha _i$. In the original model, the position of the cell was governed by the ordinary differential equation

$$\begin{aligned} C_d \frac{\mathrm {d} \mu }{\mathrm {d} t} = \sum _{i = 1}^n -\alpha _i \left( \mu - X_i \right) \phi _i(t) \end{aligned}$$

where $C_d$ is the drag coefficient and $\phi _i(t)$ is an indicator function which takes value 1 if site *i* is attached, and otherwise 0. It was also shown (Dallon et al. 2013a) that a simplified “centroid model,” accounting only for the cell position in equilibrium, can be used to approximate the differential equation model. It is shown that it is a valid assumption when the ratio of spring coefficient to drag coefficient is large which it is for physiological ratios $\alpha /C_d$ between 24.9 and 900 $\hbox {min}^{-1}$ (Dallon et al. 2013a).

We make three further simplifying assumptions compared to the previous centroid model by Dallon. The first is that we consider the case of $n = 2$ adhesion sites. This can be regarded as a type of left-right orientation of a migrating cell in 1D, where each site represents the average behavior of all adhesions on either side of a cell. The second is that adhesion sites update positions instantaneously and do not spend time being detached. This results in our centroid model taking the form

1 $$\begin{aligned} \mu = \frac{\alpha _1 X_1 + \alpha _2 X_2}{\alpha _1 + \alpha _2}, \end{aligned}$$

where the cell migrates by updating the position of adhesion sites. Since we assume that adhesion site updates are instantaneous, so is the new nucleus position following an update of an adhesion site. The mechanism for how the new position of an adhesion site is chosen and how the cell springs $\alpha _i$ are chosen is discussed in detail in Sect. 2.3.

As the cell nucleus connects to the adhesion sites with elastic springs of rest length 0, it exerts forces on the ECM, which in turn is an elastic material. The balancing of the cell forces and the ECM forces is at the core of our model. The force exerted by the cell at adhesion site *i* is given by

2 $$\begin{aligned} F_i = \alpha _i(\mu - X_i) \end{aligned}$$

and will always point toward the center of the cell. Since the cell deforms the substrate, the equilibrium position of the cell and its adhesion sites are those where the forces exerted by the cell are balanced by the forces from the ECM. To be able to distinguish between the position of the cell and its adhesion sites in an undeformed and deformed ECM, we introduce Lagrangian (material) and Eulerian (spatial) coordinates. The position of an adhesion site in the Lagrangian description is denoted $X_i$ using uppercase letters, and the position in the Eulerian description by $x_i$ in lowercase letters. The relationship between the Lagrangian and Eulerian coordinates is provided using the displacement function *u*, which is obtained by solving the force-balance problem discussed in the next section:

3 $$\begin{aligned} x_i = X_i + u(X_i). \end{aligned}$$

The Eulerian position $x_i$ is given as the Lagrangian position $X_i$ plus displacement at that particular position $u(X_i)$. We denote by $\mu _L$ the position of the nucleus in the Lagrangian description and $\mu _E$ in the Eulerian description. Figure 2 shows a cell initially placed on an undeformed ECM and its Lagrangian position, and below it the corresponding state when the cell exerts forces on the ECM, giving the Eulerian description. We next describe our model of the ECM and then go on to describing in detail how the cell updates its adhesion sites and how the cell springs are determined.

### Model of the Extracellular Matrix

The extracellular matrix is modeled as a 1D elastic rod with fixed endpoints at $\pm L \in \mathbb {R}$. Our model of the ECM is that of a linearly elastic material and has been used to model the mechanical behavior of gels in both experimental settings (Gjorevski and Nelson, 2012) as well as in mathematical models (Escribano  et al. 2018). The stiffness of the rod is described by the nonnegative function $C(X) = A(X)E(X)$, the product of its cross-sectional area *A*(*X*) and the elastic modulus *E*(*X*), defined for $X \in [-L, L]$. In this work, we first consider a spatially homogeneous material with constant thickness resulting in constant *C*, and in the second part a material of constant thickness but linearly increasing modulus, resulting in a linear function *C*(*X*). The displacement function of the ECM displacements, $u = u(X)$, satisfies the equations (Reddy 2013)

4 $$\begin{aligned} \frac{\mathrm{d}}{\mathrm{d}X}\left( C\frac{\mathrm{d}u}{\mathrm{d}X}\right) + Q(X)&= 0 \nonumber \\ u(L) = u(-L)&= 0 \end{aligned}$$

where *Q*(*X*) describes the forces acting on the rod. The only forces we consider are coming from the cell itself, and they are point forces applied at the location of the adhesion sites. Notice that the forces depend on how far away each adhesion site is from the nucleus. When the displacement is given by *u*, the nucleus is located at $\mu _E = \frac{\alpha _1x_1 + \alpha _2x_2}{\alpha _1 + \alpha _2}$ of each adhesion site in the Eulerian description is $x_i = X_i + u(X_i)$. Therefore, the force term takes the form

5 $$\begin{aligned} Q(X) = \sum _{i = 1}^2 \alpha _i\left[ \mu _E - (X_i + u(X_i))\right] \delta (X-X_i) \end{aligned}$$

where $\delta$ is the Dirac delta distribution at the location of the adhesion sites.

**Fig. 1:**
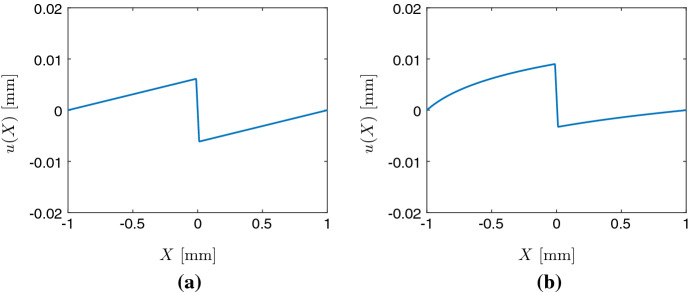
Plot of the displacement function *u* in the case of a substrate with constant stiffness (left) and linearly increasing stiffness (right). Cell size $20 \upmu$m, on domain $[-1, 1]$ mm, with $C = 1$ kPa and $C(X) = 1 + 0.8X$ kPa, respectively, $\alpha _1 = \alpha _2 = 100$ N/mm (Color figure online)

Figure 1 shows an example of the displacement function *u*, a solution to (4), for two point forces applied by the cell, both pointing toward the nucleus located at the center of the cell when $\alpha _1 = \alpha _2$. For both constant stiffness (Fig. 1a) and linearly increasing stiffness (Fig. 1b), it shows that the leftmost region is extended to the right, the middle region is compressed, and the rightmost region is extended to the left.

**Fig. 2:**
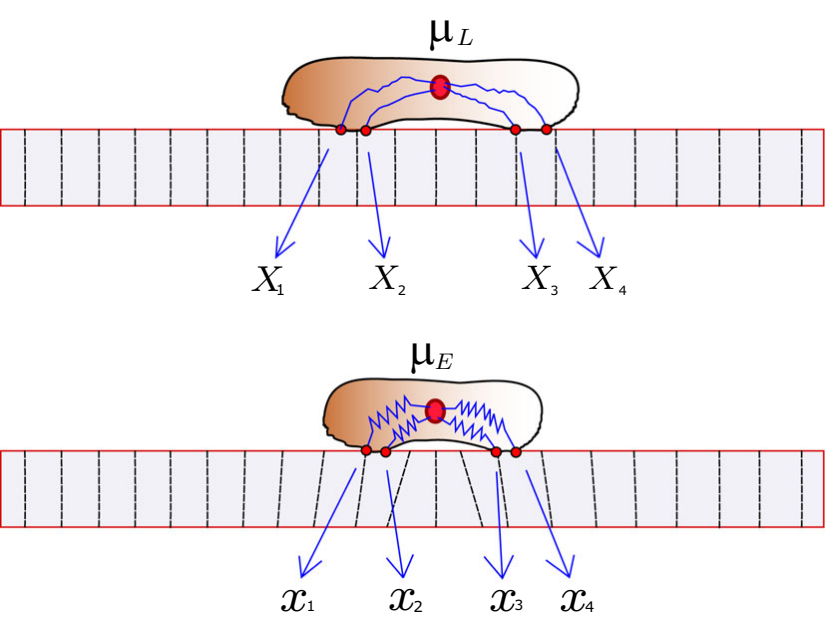
Illustration of the cell on the undeformed ECM (Lagrangian description) and the corresponding cell on the deformed ECM (Eulerian description) (Color figure online)

**Fig. 3:**
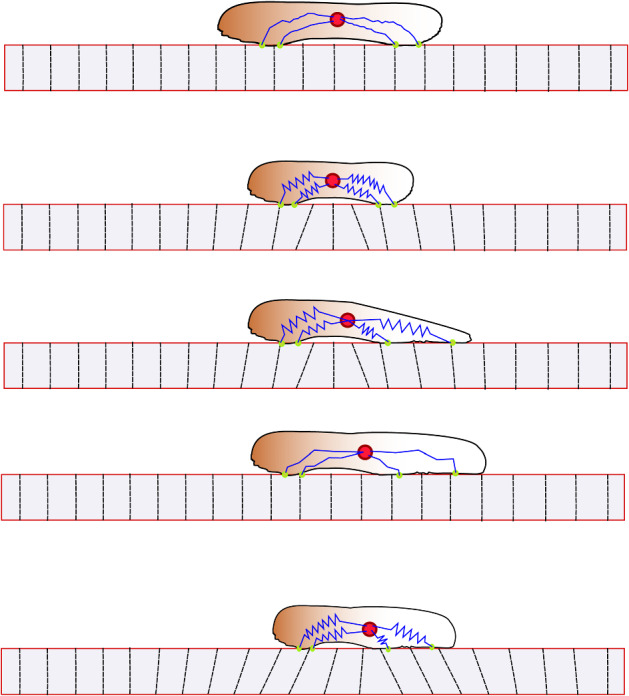
Cartoon of the steps of cell migration. (*i*) Initial state of cell on an undeformed substrate when the cell exerts no force. (*ii*) The cell exerts force so the substrate deforms. (*iii*) The cell updates the position of an adhesion site on the deformed substrate. (*iv*) We find the corresponding new position in the Lagrangian description, by relaxing the forces exerted by the cell. (*v*) Again the cell exerts force so that the substrate deforms. We are now back in the same situation as in (*ii*) (Color figure online)

### The Mechanism of Cell Migration on an Elastic Extracellular Matrix

We now go into detail of how a cell migrates through the elastic ECM. A simulation is initiated by placing a pre-strained cell onto an undeformed ECM. As the cell is placed on the ECM, it exerts forces so the ECM becomes deformed. The equilibrium position where the cell and ECM forces are balanced is found by solving (4), with the force term given by (5). These two first steps are demonstrated in Fig. 2. The time between update events is given by $\Delta t$, and once an update takes place, one of the two adhesion sites is chosen randomly. Each site has equal probability to update its position. The new position of the adhesion site is normally distributed around the current Eulerian nucleus position, with variance $\sigma ^2$. That is, the new position of an adhesion site is a random variable denoted $x_i^+$ given by

6 $$\begin{aligned} x_i^+ = \mu _E + W_E, \end{aligned}$$

where $W_E$ is a normally distributed random variable with mean 0 and variance $\sigma ^2$. This becomes the new position, expressed in Eulerian coordinates, of the adhesion site that made a jump, and a new force balance will dictate the new position of both adhesion sites. Because of the mechanical coupling between cell and substrate, when a single site makes a jump, the Eulerian position of the adhesion site that did not make a jump also changes due to the new force equilibrium. Notice that although the Eulerian position of all sites changes, the Lagrangian position of the site that did not update remains unchanged. This motivates the following procedure for computing the new equilibrium positions after a jump. Let site *i* be the site that updates its position. Its new Eulerian position is denoted $x_i^+$, and we find the corresponding Lagrangian site $X_i^+$ which satisfies

7 $$\begin{aligned} X_i^+ + u(X_i^+) = x_i^+. \end{aligned}$$

This gives us the two sites in the Lagrangian description, in which only a single site has changed. From these Lagrangian positions, we compute the new displacement by solving (4). These two new Eulerian positions are then the new positions after the jump. A cartoon figure illustrating our cell migrating on an elastic ECM is shown in Fig. 3, and an algorithmic description is provided in Algorithm 1 below. It would be desirable to find the probability distribution governing adhesion site updates in the Lagrangian description, as this would effectively make the migrating cell into a random walker as in Malik and Gerlee (2019); however, this is a non-trivial problem as the distribution would depend on both the current properties of the cell $X_1, X_2, \alpha _1, \alpha _2$ and the substrate stiffness *C* and domain size *L* in a complicated way.
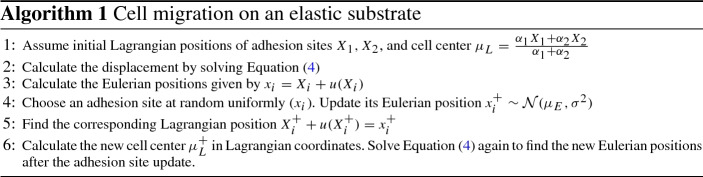


#### Plastic Properties of the ECM

As an extension of the elastic ECM model, we consider the impact of plastic deformation of the ECM. It has been demonstrated that cells can induce permanent strain in the ECM (Kim et al. 2017; Nam et al. 2016). Pairs of cells can exert forces and significantly remodel the matrix between them, forming collagen bundles consisting of aligned densified collagen fibers (Kim et al. 2017). It was also shown that the collagen bundles formed and matured on a timescale of about 30-60 minutes.

After investigating cell migration on the elastic substrate, we consider the fully plastic description of the ECM. We do this by assuming that the displacements induced by the cell traction forces remain permanently. Mathematically, this means that once the displacement ODE (4) is solved and the Eulerian positions of the adhesion sites are computed, these positions become the Lagrangian positions in the next time step, and the material never bounces back to its non-stressed configuration as in the elastic case. This is a reasonable assumption because of the fast timescale of which the cell reaches equilibrium compared to the maturation time observed in Kim et al. (2017).

### Cell Forces Depend on Substrate Stiffness

The relationship between the forces generated by migrating cells and the stiffness of the substrate is well studied (Saez et al. 2005; Han et al. 2012; Tee et al. 2011; Mitrossilis et al. 2009). Although the exact relationship between substrate stiffness and cell spreading area, adhesion dynamics and traction forces is not entirely understood, it appears that the traction forces generated by the cells are proportional to the substrate stiffness, with some upper bound on the maximum force the cells are able to generate (Mitrossilis et al. 2009). This motivates the first choice of cell spring coefficients as

8 $$\begin{aligned} \alpha _i = \kappa C(X_i) \end{aligned}$$

where $\kappa$ is a proportionality coefficient. We also compare this to the simplest nonlinear relationship, namely the case where the cell springs are quadratic function of the substrate stiffness:

9 $$\begin{aligned} \alpha _i = \kappa _0 + \kappa C(X_i)^2 \end{aligned}$$

In order to determine how these particular choices impact cell migration, we compare them to a baseline case where spring constants are equal and constant $\alpha _1 = \alpha _2 = \alpha$.

## Results

In this section, we simulate our model in order to investigate how the deformations of the ECM as well as the cell traction forces influence cell migration. To compare our simulation to the experimental observations in DuChez et al. (2019), we use the two measures introduced there, namely the forward migration index (FMI) and cell speed, defined by

10 $$\begin{aligned} \mathrm{FMI} = \frac{\mu _{\text{ end }}}{L_p} \end{aligned}$$

where $\mu _{\text{ end }}$ is the position of the cell at the end of the simulation and $L_p$ is the total distance traveled by the cell during the simulation. The average cell speed is defined as

11 $$\begin{aligned} S = \frac{L_p}{T} \end{aligned}$$

where *T* is the duration of the simulation. In all our simulation we use the spatial domain $[-1, 1]$ mm. We assume that total traction forces exerted by cells vary approximately between 50 nN - 1000 nN (Mitrossilis et al. 2009; Han et al. 2012; Tee et al. 2011) which are of a realistic magnitude, and substrate stiffness that ranges between 2 kPa and 30 kPa (DuChez et al. 2019; Tee et al. 2011; Saez et al. 2005). We choose the time between update events to be $\Delta t = 30 \text{ min }$ so that the average cell speed lies within the range observed for human cancer cells (DuChez et al. 2019), i.e., between 6 and 48 $\upmu$m/h. The variance of adhesion site jumps $\sigma ^2$ governs the typical cell size. Numerical simulations have shown (results not shown) that the typical cell size is about $0.9 \sigma$. We therefore choose $\sigma = 0.025$ mm, in order for the cells to be approximately 22 $\upmu$m in size. We perform 35000 simulations of single cells to obtain adequate statistics.

### Uniform Substrate Stiffness

We now begin by investigating cell migration in the three different cases: the first case is that of constant cell springs, and then the two cases of linear or quadratic dependence on substrate stiffness. In the case of constant cell spring coefficients, we use $\alpha _1 = \alpha _2 = 100$ nN/$\upmu$m. For the linear case, we choose

12 $$\begin{aligned} \alpha _i = 3 C(X_i), \end{aligned}$$

and for the quadratic case, we choose

13 $$\begin{aligned} \alpha _i = 0.5 + 0.5C(X_i)^2. \end{aligned}$$

We place cells on uniform substrates of varying stiffness ranging from 5 kPa to 75 kPa. We perform simulations of single cells with $\Delta t = 30 \text{ min }$ between update events, for a total duration of $T = 12$ hours. We compute the cell speed (11) as well as average ECM displacement at the location of the adhesion sites.

**Fig. 4:**
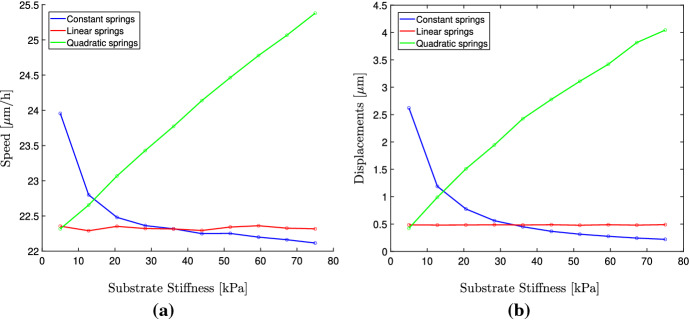
Plot of the average cell speeds for (**a**) and average ECM displacements (**b**), for varying substrate stiffness. The cell spring coefficients are equal and constant (Color figure online)

As can be seen from Fig. 4a in the case of constant cell springs, the average cell speed is higher on soft substrates than on stiff substrates. It is also the case that the average ECM displacements are larger on softer substrates, which is expected. The reason for the larger speeds on softer substrates is due to the displacements being larger. This can be explained by considering how the cell updates the position of its adhesion sites. On a substrate that is subject to large deformations, any particular distance in Eulerian coordinates corresponds to a larger distance in Lagrangian coordinates. Therefore, as a cell is migrating by repeatedly updating the position of its adhesion sites in Eulerian coordinates on a highly deformed ECM, the movement of the cell in Lagrangian coordinates is larger resulting in faster migration speeds. This stands in contrast to observations from the literature, where it is typically the case that cells spread out faster on stiff matrices or show a biphasic relationship where the optimal migration speed is obtained for intermediate matrix stiffness (DuChez et al. 2019; Pathak and Kumar 2012; Zaman et al. 2006; Lang et al. 2015). In the case where the cell springs depend linearly on the substrate stiffness, we can see that the average substrate deformations remain the same independent of substrate stiffness, which has been observed experimentally (Saez et al. 2005). Because of this, the cell migration speed is also constant and independent of the substrate stiffness. This assumption therefore results in more realistic migratory behavior than assuming constant spring coefficients. Finally, we can see that when using a quadratic stiffness function, the cells do migrate faster on stiffer matrices. This is the results of the displacement being much larger than in the case of the constant or linear relationship. However, for substrate stiffness larger than about 30 kPa the displacements become unrealistically large.

The FMI is close to 0 in all cases (results not shown), which is expected on a substrate of uniform stiffness.

### Migration in the Presence of a Stiffness Gradient

In this section, we use the linearly increasing stiffness function

14 $$\begin{aligned} C(X) = 12.5 + 10.5X \end{aligned}$$

**Fig. 5:**
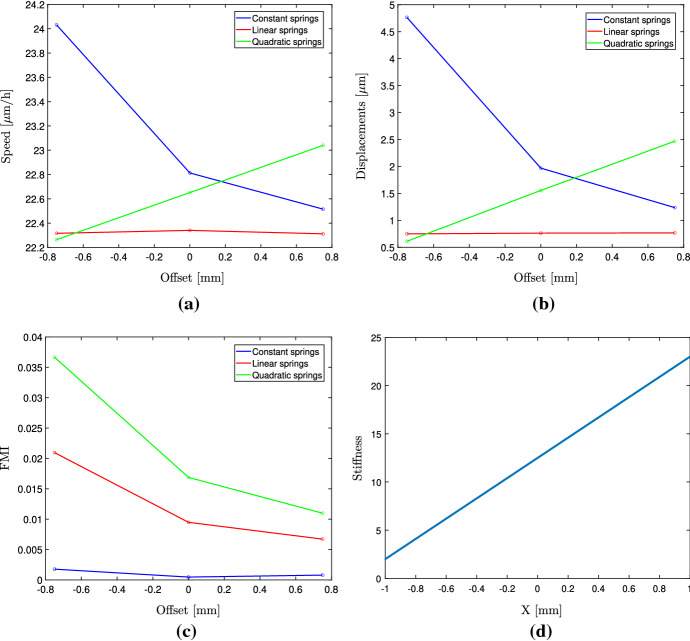
Plot of the average cell speeds for (**a**) and average ECM displacements (**b**), FMI (**c**) and average cell position (**d**) for varying cell spring coefficients $\alpha$. The stiffness function with a gradient is shown in (**d**) (Color figure online)

which on the domain $[-1, 1]$ mm results in a stiffness in the range of 2 kPa up to 23 kPa, to match the one in DuChez et al. (2019) (Fig. 5d). Our numerical experiment consists of placing cells on the domain $[-1, 1]$ mm, with different starting positions. They start with an offset from the origin of either $-0.75$, 0 or $0.75 \text{ mm }$ from the origin. This corresponds to cells being placed in either the soft (2-7 kPa) region, the medium region (7-13 kPa) or the stiff region (13-18 kPa). We wish to compare our simulations to the experiment conducted in DuChez et al. (2019), where it was shown that cells seeded on the soft region (2-7 kPa) showed the largest FMI, compared to medium (7-13 kPa) and stiff (13-18 kPa) regions. On soft regions the FMI for the four cell types ranged between about 0.1 and 0.17, for the medium region between 0.035 and 0.07, and on the stiff region below 0.05. It was argued that this could be the result of the stiffness gradient being relatively larger in the soft region than the stiff regions.

We begin by investigating the case of constant cell spring coefficients $\alpha _1 = \alpha _2 = 100$ nN/$\upmu$m. The resulting speeds, displacements and FMI are shown in Fig. 5. As can be seen, the migration speeds as well as the displacements are lower on the stiff and medium region compared to the soft region. The FMI is close to 0 in all three regions.

We now again use the linear function for stiffness-dependent cell spring coefficients, given by Equation (12) along with the stiffness function given by (14). The results are shown in Fig. 5. As expected, the average cell speed and the average ECM displacements are independent of the starting position. From Fig. 5c, we can see that the FMI is indeed largest on the soft region and decreases as the local stiffness at the starting position increases. Our results agree well qualitatively with those of DuChez et al. (2019); however, our FMI values are about one-fifth of those reported in DuChez et al. (2019). We can see that the average cell speed remains the same on all three regions, which is in agreement with the experimental analysis where it is concluded that cell speed does not contribute to the observed migration up the stiffness gradient. When comparing constant cell springs with stiffness-dependent cell springs, it is clear that substrate-dependent springs result in realistic behavior, whereas constant cell springs do not capture the behavior of the migrating cells showing a strong durotactic response.

We finally use the quadratic relationship and see that it captures both the phenomena of increased cell migration on stiffer regions (Fig. 5a) and the large FMI on the soft region (Fig. 5c). The magnitude of the FMI shows a better quantitative agreement with the experimental observations compared to the linear relationship.

### Plastic ECM Model

**Fig. 6:**
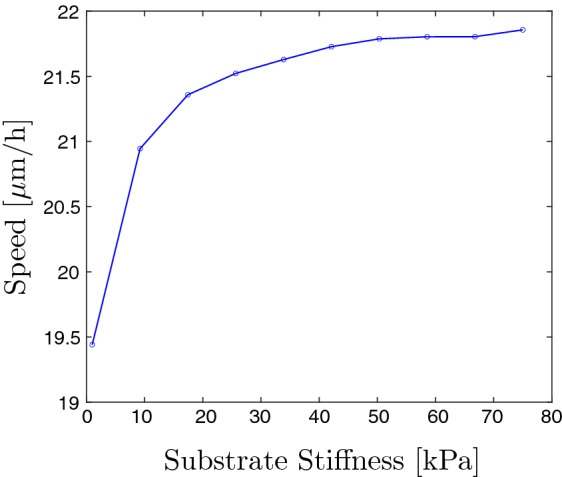
The cell migration speed of a migrating cell in a plastic EMC of varying stiffness (Color figure online)

We now consider the fully plastic ECM model described in Sect. 2.3.1. We perform numerical simulations with constant cell springs and all parameters being the same as for the case of constant cell springs in an elastic ECM in Sect.  3.1. We investigate particularly the impact on cell migration speed. The results are shown in Fig. 6. It can be seen that the cell speed is increasing with the substrate stiffness, up to a point where it reaches a plateau. On very soft substrates, where the displacements of the plastic EMC are large, the cell cannot migrate at full speed, as the large displacements hinder movement. Large displacements in the elastic model result in large migration speeds due to the substrate bouncing back to its stress-free configuration. However, when this does not occur, the large displacements have the effect of decreasing the migration speeds.

## Discussion

The mechanical properties of the ECM are known to influence the behavior of cells in many ways, in particular cell migration. In this study, we have developed a mathematical model to investigate the impact of elastic deformations on cell migration under the assumption that cells move randomly, with no preference for a particular direction. The model assumes that cells migrate by updating the position of one of its adhesion sites. The cell is coupled to the ECM and exerts forces on the substrate which leads to ECM deformations, which in turn affects the location of adhesion sites on the deformed substrate.

We have carried out a systematic investigation of how the stiffness impacts migration in the case of both a uniform stiffness profile and a linearly increasing one. We differentiated between three mechanisms for the cells to generate forces: the first where the cell spring coefficients are constant, the second where the cell spring coefficients depend linearly on the substrate stiffness, and the third where they depend quadratically on the substrate stiffness. The mechanisms where the cell springs are dependent on the substrate stiffness are more realistic, as it is known that cells typically generate larger traction forces on stiffer substrates. Comparing the three cell force mechanisms on uniform substrates, we show that in case of using constant cell spring coefficients, the model shows that cells migrate faster on soft substrates, which is contrary to most experimental observations. This is the result of larger deformations and hence a larger discrepancy between Lagrangian and Eulerian coordinates. As a soft substrate is deformed to a greater extent, the migration speed will increase too. This is not the case when the more realistic stiffness-dependent form is used. When we introduce a stiffness gradient, it is clearly shown that substrate-dependent cell forces result in much stronger durotactic motion up the stiffness gradient. Our model manages to capture the qualitative behavior observed in DuChez et al. (2019), where all four cell types show a much stronger durotactic response when the stiffness gradient is large compared to the local stiffness. In the three regions considered here, this ratio between gradient and local stiffness ranges from about $1.94 \text{ mm }^{-1}$ in the soft region, to $1.19 \text{ mm }^{-1}$ and $0.44 \text{ mm }^{-1}$ in the medium and stiff regions, respectively. This was also observed in a previous computational model (Escribano  et al. 2018); however, the elastic deformations were not studied in isolation, but in combination with cell growth. Our model exhibits this behavior as a result of the gradient appearing larger to the cell when the local stiffness is lower. The result that the average cell speed is independent of which region the cells are seeded into support this, and hence, we have shown that the durotactic response in our model is indeed not the result of varying cell speeds in the different regions; however, in the case of a quadratic relationship, the speed is increased as well as the durotactic response. Our results therefore seem to support the notion that it is the ratio of stiffness gradient to absolute stiffness that is driving durotaxis. This was the case also in Malik and Gerlee (2019) where the lifetime of adhesion sites was reinforced on stiff substrates, and two different sensing mechanisms were investigated. The first is that the cell can sense the relative difference in substrate stiffness at the different adhesion sites which then determines the lifetime of the sites. The second is that it is the absolute difference in stiffness that governs the lifetime of the sites. It was shown that these two mechanisms resulted in movement up the gradient that was proportional to either $C'(X)/C(X)$ or $C'(X)/C^2(X)$, respectively.

Lastly, we also investigate a fully plastic model of the ECM, where the strains induced by the cells remain permanently. We demonstrate that this assumption, along with constant cell spring coefficients, results in decreased migration speeds on soft substrates.

Our proposed model is a much simplified description of cell migration. To make the analysis tractable, we have assumed that the single cell migration takes place on a 1D ECM, and that each cell only has two adhesion sites. The model is not constructed to make quantitative predictions, but rather to investigate the qualitative aspects of cell migration on an elastic substrate. It shows that the assumption of constant cell spring coefficients does not suffice to explain the durotactic motion, or the impact of stiffness on migration speed. In particular, to observe any significant amount of durotaxis, the displacements of the ECM need to be in a range which is not realistic. On the other hand, when assuming that the cell can generate greater traction forces on stiff substrates, the model shows the same qualitative behavior as has been observed for human cancer cells (DuChez et al. 2019).

Although durotaxis has been modeled previously, in many cases it is done by explicitly modeling how durotaxis is expressed, i.e., assuming that it will occur, e.g., through movement up the gradient being more likely (Stefanoni et al. 2011; van Oers et al. 2014), or by directly influencing cell speed and turning rate (Doering et al. 2018), or persistence (Novikova et al. 2017). Although very useful in quantitative predictions, they offer less insight into the mechanisms driving durotaxis. The influence of ECM deformations on cell migration has been modeled previously in Escribano  et al. (2018), and it was concluded that deformation is sufficient to observe a significant durotactic response. However, they also model cell growth, which is dependent on substrate stiffness, and hence do not isolate the impact of deformations. Our study offers such insight into how elastic deformations influence directed cell migration, and how force generation needs to be dependent on stiffness in order for the cells to efficiently migrate up a gradient.

The proposed model could be extended in a number of ways. The first would be to consider other models of the ECM and its material properties, to be able to model a wider range of substrates in both 2D and 3D. Although a linearly elastic model comes as a natural first choice, it is likely that choosing another model of the substrate’s response to forces could result in new results. In particular, when modeling collective cell migration where multiple cells are seeded onto the same substrate, cells exert forces which may be sensed by neighboring cells. In the current framework, displacements occur along the full substrate length, whereas a more realistic model would permit only finite range displacements within a close proximity of each cell. Although experimental studies indicate that the traction forces appear to be proportional to the substrate stiffness, there must exist an upper bound on the magnitude of the forces generated by cells. Therefore, one can choose a more realistic relationship between the cell spring coefficients and the substrate stiffness. Such a choice could be any S-shaped function with an upper bound.

In Malik and Gerlee (2019) we investigated the impact of stiffness-dependent adhesion lifetimes on cell migration. It was shown that under the assumption that adhesion sites become reinforced and remain longer on stiff regions, cells migrated up a stiffness gradient. This assumption could be combined with the one from the current study, to model traction forces which depend on the local stiffness, where the lifetime of adhesions depends on the force generated by the cell. These two mechanisms combined should result in a durotactic response that is stronger than the two mechanisms in isolation.
